# Further Validation of the Norwegian* Self-Efficacy for Therapeutic Mode Use*

**DOI:** 10.1155/2017/9745373

**Published:** 2017-09-10

**Authors:** Victoria C. Ritter, Mikkel M. Thørrisen, Farzaneh Yazdani, Tore Bonsaksen

**Affiliations:** ^1^Department of Occupational Therapy, Prosthetics and Orthotics, Faculty of Health Sciences, Oslo and Akershus University College of Applied Sciences, Oslo, Norway; ^2^Faculty of Health and Life Sciences, Oxford Brookes University, Oxford, UK; ^3^Faculty of Health Studies, VID Specialized University, Sandnes, Norway

## Abstract

**Background:**

The Intentional Relationship Model (IRM) proposes six therapeutic modes as ways of relating to clients. The Norwegian* self-efficacy for therapeutic mode use* (N-SETMU) was found to have a one-component structure. However, its items reflect abstract concepts rather than concrete behaviors.

**Aim:**

To validate further the N-SETMU by linking its items to the Norwegian* client assessment of modes* (N-CAM), with 30 items constituting six scales (linked to each mode), possessing concrete, behavioral content.

**Methods:**

Occupational therapy students (*n* = 111) completed the N-SETMU and the N-CAM derived items, along with sociodemographic information. Component structure was analyzed with Principal Components Analysis (PCA), internal consistency of scales with Cronbach's *α*, and associations between scale scores with Pearson's *r*.

**Results:**

All items on all N-CAM derived scales loaded on one latent component, except one item related to* problem-solving*. After removing this item, the scale functioned appropriately. Cronbach's *α* for all N-CAM derived scales ranged 0.88–0.94, and the associations between the N-CAM derived scales and the corresponding N-SETMU items ranged between 0.60* (advocating)* and 0.79* (encouraging)*.

**Conclusions:**

In view of the strong associations between the concrete, N-CAM derived scales and the abstract N-SETMU items, this study supports the concurrent validity of the N-SETMU.

## 1. Introduction 

Self-efficacy refers to a person's belief that he or she has the capabilities to organize and execute necessary actions to produce given attainments [1, 2]. Once formed, self-efficacy influences the person's choice of action and course of behavior. The concept is used to explain what people choose to endeavor, the amount of effort they invest into the endeavor, and the tenacity they show when doing it. When people believe that they can produce desired effects by their own doing, they may put forth a greater effort and have more incentive to persevere when faced with adverse situations or obstacles. When setbacks occur, persons with higher self-efficacy may recover more quickly and remain committed to their goals. Empirically, persons with higher self-efficacy for a specific task have a greater chance of actually succeeding at it, as demonstrated in a range of studies. For example, among adolescents with diabetes, self-efficacy for dieting has been associated with improved adherence to a recommended diet [3]. Similarly, self-efficacy for smoking cessation has been associated with actual stop smoking [4], and self-efficacy for academic performance has been associated with better actual performance among advanced higher education students [5].

As self-efficacy is believed to influence a person's motivation for and success in performing occupations [1], the concept has gradually been incorporated into occupational therapy research. Self-efficacy in occupational therapy clients is considered crucial for their goal attainment and treatment outcomes. However, self-efficacy for a range of different behaviors and skills is needed also for successful work performance among occupational therapists themselves. Vax and coworkers [6] assessed 64 occupational therapists working in mental health in Israel and found that higher levels of work-related self-efficacy were significantly associated with higher age, more seniority, higher level of education, and higher level of general self-efficacy.

Of specific importance for occupational therapists' success in clinical practice is their capacity for building and maintaining a positive relationship with clients [7]. To be able to foster such a relationship with clients, occupational therapists' ability to use different therapeutic approaches as appropriate to clients' needs is vital. However, managing the relational aspects of therapy with the appropriate use of therapeutic approaches also depends on the therapist's ability to recognize the client's interpersonal behaviors and preferences and the therapist's ability to transform these observations into action. Thus, occupational therapists' use of self in therapeutic practice is multifaceted [7]. It requires the therapist's observational skills for recognizing the client's characteristics, and it requires skills in using different therapeutic approaches. It also requires the therapist to adapt his or her therapeutic approach carefully and judiciously based on the situation at hand. To be successful in therapeutic practice, according to Bandura's [1] reasoning, the occupational therapist would ultimately need self-efficacy for coping with all these relational challenges that, to varying degrees, are inherent in all client-therapist relationships.

Instruments for assessing self-efficacy for therapeutic use of self should be examined systematically in order to gain knowledge of their psychometric properties. To date, one such study has been performed with the Norwegian version of the* self-efficacy for therapeutic mode use* (N-SETMU). Bonsaksen and Carstensen [8] examined the properties of this instrument and found that the items had a one-component structure and a high level of internal consistency. They noted, however, “respondents are required to have a basic conceptual understanding of the practical content of each mode in order to provide valid responses [to the N-SETMU]” (p. 3). Further, they suggested that future studies might measure self-efficacy for mode use with items that describe specific and concrete mode behaviors as an alternative to items referring to the abstract mode descriptor itself. The need to examine whether the participants' self-efficacy for mode use is consistent with their self-efficacy for the concrete behaviors typical for each mode constitutes the rationale for the present study.

*Study Aim*. The aim of the study was to contribute further to the validation of the N-SETMU, a recently established assessment tool for measuring self-efficacy for therapeutic mode use among occupational therapists. Specifically, we aimed to assess the strength of the associations between the Norwegian* client assessment of modes* (N-CAM) scales based on concrete, behavioral items, and the corresponding N-SETMU item.

## 2. Materials and Methods

### 2.1. Design and Context

The study had a cross-sectional design. It was conducted at the occupational therapy education programs in Oslo and Trondheim, which are both three-year full-time undergraduate programs.

### 2.2. Participant Recruitment

Participants in the study were occupational therapy students in their second year of study. The students were included as participants based on their (a) enrolment in one of the relevant education programs and their (b) provided informed consent to participate. The questionnaires were completed approximately four months after the students' participation in IRM workshops at each of the sites.

### 2.3. Measures

The* self-efficacy for therapeutic mode use* (SETMU) was developed by Yazdani and Tune in the United Kingdom and was subsequently translated into Norwegian [8]. The translation into Norwegian was followed by back-translating into English by a person proficient in Norwegian and English. Subsequently, Dr. Yazdani checked the content of the back-translated version for correctness and conceptual clarity by comparing it with the original version, after which no further changes were required.

When completing the Norwegian version of the instrument (N-SETMU), respondents rate their level of confidence that they possess the required skills to use each of the therapeutic modes in client-therapist encounters. The therapeutic modes, described in the IRM as the advocating, collaborating, empathizing, encouraging, problem-solving, and instructing modes [7], are listed as items on the N-SETMU. For all items, respondents rate their level of confidence on a 1–10 scale, 1 indicating the lowest possible level of confidence and 10 indicating the highest possible level.

The* client assessment of modes *(CAM) was developed by Taylor and coworkers [9, 10] in the USA and subsequently translated into Norwegian. The Norwegian therapist version of the instrument (N-CAM) was used in this study. The translation procedure was similar to, but also more extensive, than the procedure described for the N-SETMU. Two independent forward translations were developed for the N-CAM, which were both discussed within a research group and revised before the two initial versions were merged into one agreed-upon version to be sent to the instrument developer. Professor Taylor checked the content of the harmonized back-translated version for correctness and conceptual clarity in light of the original CAM, and, following minor revisions, the N-CAM was deemed to reflect well the contents of the original assessment.

For the purpose of the present study, the N-CAM items were modified to focus on the respondent's self-efficacy for the behavior addressed in each item. For example, item 1 in the original CAM reads, “I helped the patient get access to resources or people in the community in which he or she lives” [9]. In the present study, this item read “if appropriate for the situation, I am confident in my ability to help the patient get access to resources or people in the community in which he or she lives.” An overview of example items related to each of the six modes is provided in Table 1. For all items derived from the N-CAM, respondents rated their level of confidence on a 1–10 scale, 1 indicating the lowest level of confidence and 10 the highest level of confidence. Information regarding the participants' age and gender was collected by the time of study recruitment.

### 2.4. Data Analysis

All data were analyzed the computer program IBM SPSS [11], and statistical significance for all analyses was set at* p* < 0.05. First, a Principal Component Analysis (PCA) was performed. The modified N-CAM items were entered into the analysis in six subsequent sections, according to their proposed structure, that is, according to the mode each item belongs to [9]. Component extraction was determined by visual inspection of the scree plot and by assessing the eigenvalue (*λ*) estimates. According to statistical convention [12, 13], component extraction was performed if *λ* > 1. In the eventual case of extracting more than one component in each section of the analysis, the direct oblimin rotation method was to be used in order to obtain a clearer structure matrix. In addition to the *λ* estimates, the statistical measures reported from the analysis include communalities, which are each item's variance proportion explained by the factors together, and the component loadings, which are estimates of the impact from a given item on each component. Component loadings > 0.40 were considered high.

Second, the internal consistency of the resulting scales was examined with Cronbach's *α*. Internal consistency estimates vary with the number of items belonging to a scale and with the size of the sample yielding the data [14, 15]. Cronbach's *α* > 0.70 is usually considered good, and this was used as our criterion.

Third, associations between the N-SETMU items (i.e., self-efficacy for using each mode) and the N-CAM scales were assessed with Pearson's correlation coefficient* r*. Strong positive correlation coefficients [*r* > 0.50, [16]] would be interpreted as supporting the concurrent validity of the N-SETMU. Such associations would indicate strong relationships between self-efficacy for the concrete behaviors encompassed by each therapeutic mode (measured with the N-CAM) and self-efficacy for using the corresponding therapeutic mode, described as such (measured with the N-SETMU).

### 2.5. Ethics

Approval for conducting the study was obtained from the Norwegian Data Protection Official for Research (project number 49433). The students were informed that completion of the questionnaires was voluntary, that their responses would be treated in confidence, and that there would be no negative consequences from opting not to participate in the study. Written informed consent was provided from all participants.

## 3. Results

### 3.1. Participants

The participants in this study were 111 occupational therapy students from the second study year, enrolled in education programs in Oslo (*n* = 47) and Trondheim (*n* = 64), respectively. The mean age of the students was 24.5 years (SD = 6.0 years). Female students were in majority within the subsamples from both universities (Oslo *n* = 37, 78.7%; Trondheim *n* = 51, 79.7%). There were 142 students enrolled in the relevant cohorts of the two education programs, yielding a response rate of 78.2%. Among the nonresponders (*n* = 31), the mean age was 23.9 years (SD = 5.2 years) and they were 29 (93.5%) women and 2 (6.5%) men.

### 3.2. Component Structure and Internal Consistency

The results related to the N-CAM items' component structure and internal consistency are displayed in Table 2. All scales fit best with a one-component solution, as for each scale, only one component satisfied the criterion of having an eigenvalue > 1. The one-component solutions explained between 67.3% (advocating) and 80.2% (encouraging) of the variance related to the relevant items. Component loadings ranged 0.75–0.87 for the advocating items (communalities 0.56–0.76), 0.86–0.92 for the encouraging items (communalities 0.74–0.85), 0.82–0.90 for the empathizing items (communalities 0.67–0.81), 0.87–0.90 for the instructing items (communalities 0.75–0.81), and 0.83–0.91 for the collaborating items (communalities 0.69–0.82). Internal consistency of these scales ranged between 0.88 (advocating) and 0.94 (encouraging).

Item 26, a part of the problem-solving mode on the CAM scale, had low communality (0.06), it did not load appropriately on the scale (loading 0.25), and the scale's internal consistency increased substantially when removing it (from 0.50 to 0.90). Thus, the scale functioned better after removing item 26, with component loadings ranging 0.84–0.93 (communalities 0.70–0.87; see Table 2).

### 3.3. Associations between CAM Scales and the Corresponding N-SETMU Items

The correlation analyses showed positive, strong, and statistically significant associations between all the N-CAM scales (with item 26 being removed from the problem-solving scale) and the corresponding N-SETMU items (all *p* < 0.001). The correlation between the advocating N-CAM scale and the advocating N-SETMU item was *r* = 0.60. Similarly, the strength of the other N-CAM scale/N-SETMU item associations was *r* = 0.61 (instructing), *r* = 0.65 (problem-solving, empathizing, and collaborating), and *r* = 0.79 (encouraging).

## 4. Discussion

One previous study suggested the N-SETMU to be exposed to further validation procedures [8], and this study addressed its concurrent validity by investigating the associations between the N-CAM scale scores and the corresponding N-SETMU item scores. Overall, the results showed that the N-CAM scales were strongly related to the corresponding N-SETMU items, thus indicating concurrent validity of the N-SETMU.

Item 26 of the N-CAM was found to be problematic in the present study sample, as it did not load on the problem-solving scale as expected. The item reads “if appropriate for the situation, I am confident in my ability to help the patient consider many different ways of doing things.” The item may be considered more complicated than the other items on the scale, as it may require the patient to consider options without the therapist asking leading questions. Thus, self-efficacy for this particular skill may require the ability to ask mind-opening questions without leading the patient towards predetermined answers to them. At the time of assessment, the students may have experienced less self-efficacy for this particular skill compared to the others. Alternatively, item 26 is the only item on the problem-solving scale that specifies that the therapist helps the patient to consider not one but* many* different ways of doing things. This item qualification may also have led to a different response pattern for this item compared to the other items on the problem-solving scale. In comparison, Fan and Taylor [10] found two other items (item 8 on the instructing scale and item 18 on the advocating scale) to depart from the main pattern of unidimensionality within each of the scales. Taken together, the findings indicate that response patterns may vary between samples and settings, such that measurement properties should continue to be reported for the specific samples with which these scales are employed.

Cronbach's coefficient *α*, a measure of the internal consistency of items belonging to a scale, is largely dependent on the number of scale items [14, 15]. Scales with more items generally produce higher *α* estimates. Internal consistency should exceed 0.70 for the scale to be considered reliable, whereas estimates exceeding 0.90 may indicate that there is little variation between the items on the scale, that is, the situation may point towards item redundancy rather than item homogeneity [17]. Considering the very high (>0.90) internal consistency estimates for most N-CAM scales and given that the scales were comprised of only five items each, there may be reason to treat the scales with some caution. The participants may have been unable to discriminate fully between the scale items and their content. Alternatively, the students may have responded to the items in a rather haphazard, half-automatic way while completing the questionnaire. If they did, this may have produced a similar result.

Kielhofner [18, p. 29], explaining the concept of concurrent validity, stated that “an instrument designed to capture a variable should show an association with another variable that is theoretically expected to be related to it.” In line with the above explanation, we expected the scores on the N-CAM scales and the corresponding N-SETMU items to be intrinsically related. The analysis showed that the relevant associations were all strong and statistically significant. Therefore, as the scores on the each of the measures reflect well the scores on the other measure, the findings indicate concurrent validity of the N-SETMU.

### 4.1. Methodological Considerations

The sample was relatively small and homogenous (79.3% women, mean age 24.5 years), and a convenience sample was used. Therefore, generalizing to the larger population of occupational therapy students across geographical distances and cultural contexts may not be warranted. However, the age and gender distribution largely reflect the distributions found in previous studies of occupational therapy students [19, 20]. Recruiting participants from two higher education institutions adds to the external validity of the results.

The associations between the abstract N-SETMU item scores and the more behavioral N-CAM scale scores are interpreted as indications of concurrent validity. We note, however, that although the N-CAM items are concrete, they might be seen as not entirely reflecting the behavioral level. For example, item 11 (see Table 1) not only refers to self-efficacy for what the therapist can do but refers also to the anticipated effects of the therapist's actions (that the patient feels confident about his or her doing). Finally, self-efficacy for therapeutic mode use is not synonymous with actual skills in using the modes. Thus, there may well be a discrepancy between a person's self-efficacy scores and his or her actual skills in using the therapeutic modes in client-therapist interactions.

## 5. Conclusion

In this study, the pattern of responses to each of the N-CAM derived scales indicated that the items belonged to one underlying component and that the scale items had high internal consistency. Further, strong associations were found between the concrete, N-CAM derived scales, and the abstract N-SETMU items. Based on these findings, the study supports the concurrent validity of the N-SETMU, presupposing that respondents have received appropriate education and are well familiar with the conceptual content of each of the therapeutic modes. The results suggest that the N-SETMU, in fact, measures what it intends to measure, and they contribute to the further validation of this assessment tool. Further research is needed to see whether higher self-efficacy for therapeutic mode use is associated with better clinical performance in actual practice, and whether the tool is sensitive to changes that would be expected to occur during the course of education.

## Figures and Tables

**Table 1:** Examples of items related to each of the six therapeutic modes assessed with the Norwegian client assessment of modes, adapted for the purpose of this study.

Therapeutic modes	Examples of items
	“If appropriate for the situation, I am confident in my ability to…”
Advocating	9. Talk with the patient about legal rights for people with disabilities
Problem-solving	12. Explain different choices when guiding the patient to make a decision
Instructing	8. Tell the patient how to improve his/her performance or behavior
Encouraging	11. Make the patient feel confident about what he/she is doing
Empathizing	7. Ask questions that make the patient feel comfortable talking
Collaborating	10. Make sure that the patient works on what matters most to him/her

*Note*. All items are rated on a scale from 1 (“I cannot do this at all”) to 10 (“I am very confident I can do this”).

**Table 2:** One-component solution for the scales derived from the Norwegian client assessment of modes (adapted for the purpose of this study): items, component loadings, eigenvalue estimates (*λ*), internal consistency estimates, and explained variance (*n* = 111).

Advocating		Encouraging		Empathizing		Instructing		Collaborating		Problem-solving	
Item	Loading	Item	Loading	Item	Loading	Item	Loading	Item	Loading	Item	Loading
1	0.87	16	0.92	2	0.90	27	0.90	23	0.91	12	0.93
24	0.86	25	0.90	29	0.89	15	0.90	19	0.90	30	0.88
28	0.84	5	0.90	7	0.89	3	0.88	14	0.88	4	0.87
9	0.77	21	0.89	13	0.88	22	0.87	10	0.87	17	0.84
18	0.75	11	0.86	20	0.82	8	0.87	6	0.83	26^*∗*^	—
*λ*	3.36	*λ*	4.01	*λ*	3.82	*λ*	3.88	*λ*	3.86	*λ*	3.11
*α*	0.88	*α*	0.94	*α*	0.92	*α*	0.93	*α*	0.93	*α*	0.90
Explained variance	67.3%	Explained variance	80.2%	Explained variance	76.4%	Explained variance	77.6%	Explained variance	77.1%	Explained variance	77.8%

*Note. *Results are from the exploratory Principal Component Analysis, with component extraction criterion *λ* > 1. Internal consistency results (Cronbach's *α*) are from the scale reliability analysis. ^*∗*^Item 26 was removed from the scale due to low loading on the component, and the results for this scale are after this item was removed from the analysis.
